# Association of the C47T polymorphism in superoxide dismutase gene 2 with noise-induced hearing loss: a meta-analysis^☆^

**DOI:** 10.1016/j.bjorl.2016.01.008

**Published:** 2016-04-19

**Authors:** Jing Wang, Jun Li, Kang Peng, Zi-Ying Fu, Jia Tang, Ming-Jian Yang, Qi-Cai Chen

**Affiliations:** a Central China Normal University, School of Life Sciences and Hubei Key Lab of Genetic Regulation and Integrative Biology, Wuhan, China; b Dali University, School of Basic Medicine, Dali, China; c Dali University, School of Clinical Medicine, Dali, China

**Keywords:** Superoxide dismutase gene 2, Polymorphism, Noise-induced hearing loss, Meta-analysis, Gene da superóxido dismutase 2, Polimorfismo, Perda auditiva induzida por ruído, Metanálise

## Abstract

**Introduction:**

Currently, there is limited information about the relationship between manganese superoxide dismutase (sod2) c47t polymorphism and susceptibility to noise-induced hearing loss (NIHL).

**Objective:**

The aim of this meta-analysis was to clarify the association between SOD2 C47T polymorphism and NIHL.

**Methods:**

A search in PubMed and Web of Science was performed to collect data. All full-text, English-written studies containing sufficient and complete case-and-control data about the relationship between SOD2 C47T polymorphism and NIHL were included. Three eligible studies, comprising 1094 subjects, were identified. pooled odds ratios (ORs) and 95% confidence intervals (CI) were calculated to evaluate the strength of the association between SOD2 C47T polymorphism and NIHL.

**Results:**

No significant association between C47T polymorphism and risk of NIHL was found with the following combinations: T *vs*. C (OR = 0.83; 95% CI = 0.63–1.09); TT *vs*. CC (OR = 0.49; 95% CI = 0.22–1.09); CT *vs*. CC (OR = 0.54; 95% CI = 0.25–1.17); TT *vs*. CC + CT (OR = 0.82; 95% CI = 0.50–1.32); CC *vs*. TT + TC (OR = 0.49; 95% CI = 0.23–1.04). However, in subgroup analysis, a significant association was found for TT *vs*. CC + CT (OR = 0.77; 95% CI = 0.42–1.41) in the Chinese population.

**Conclusion:**

The present meta-analysis suggests that SOD2 C47T polymorphism is significantly associated with increased risk of NIHL in the Chinese population. Further large and well-designed studies are needed to confirm this association.

## Resumo

**Introdução:**

Atualmente, são limitadas as informações acerca da relação entre o polimorfismo C47T de superóxido dismutase 2 (SOD2) dependente de manganês e suscetibilidade à perda auditiva induzida pelo ruído (PAIR).

**Objetivo:**

O objetivo desta metanálise foi esclarecer a associação entre o polimorfismo C47T de SOD2 e PAIR.

**Método:**

Foi realizadas buscas no PubMed e Web of Science para coleta de dados. Foram incluídos todos os estudos no idioma inglês, contendo dados suficientes e completos de casos e controles sobre a relação entre o polimorfismo C47T de SOD2 e PAIR. Foram identificados três estudos qualificados, abrangendo 1.094 indivíduos. Foram calculadas as razões das chances (*odds ratio*, OR) acumuladas e intervalos de confiança (IC) de 95% para que fosse avaliada a potência da associação entre o polimorfismo C47T de SOD2 e PAIR.

**Resultados:**

Não foi encontrada uma associação significativa entre o polimorfismo C47T de SOD2 e risco de PAIR com as seguintes combinações: T *vs.* C (OR = 0,83, IC 95% = 0,63-1,09); TT *vs.* CC (OR = 0,49, IC 95% = 0,22-1,09); CT *vs.* CC (OR = 0,54, IC 95% = 0,25-1,17); TT *vs.* CC + CT (OR = 0,82, IC 95% = 0,50-1,32); CC *vs.* TT + TC (OR = 0,49, IC 95% = 0,23-1,04). Contudo, na análise de subgrupo, foi encontrada uma associação significativa para TT *vs.* CC + CT (OR = 0,77, 95% CI = 0,42-1.41) na população chinesa.

**Conclusão:**

A presente metanálise sugere que o polimorfismo C47T de SOD2 demonstra associação significativa com maior risco de PAIR na população chinesa. Há necessidade de novos estudos de grande porte bem concebidos, para confirmação dessa associação.

## Introduction

Noise-induced hearing loss (NIHL), one of the most common occupational diseases, is a form of sensorineural hearing impairment caused by the interaction between environmental factors (such as prolonged exposure to high levels of noise) and genetic factors.^1^ According to statistical data, about one-third of all cases of hearing loss can be attributed to noise exposure,^2^ and 10% of the world's population are at risk of developing NIHL.^3^

Currently, little is known about gene polymorphisms that may be involved in the susceptibility to NIHL. Ohlemiller et al.^4^ demonstrated that noise can damage the cochlear sensorial epithelium by inducing the local release of free radicals. Consequently, genes involved in the regulation of releasing of free radicals were examined,^5^ and manganese superoxide dismutase (SOD2) was identified.^6^

SOD2 is a homotetramer located within the mitochondrion and is an enzyme involved in the conversion of superoxide radicals to hydrogen peroxide.^7^ Among the polymorphisms identified in the SOD2 gene, C47T is the most widely studied. C47T is located at position 16 in the mitochondrial targeting sequence and results in the replacement of an alanine with valine (V16A).^8^, ^9^ C47T has been studied in association with several diseases (heart disease,^10^ diabetes,^11^ and nonalcoholic fatty liver disease [NAFLD])^12^ which include NIHL.^13^ Fortunato et al.^6^ previously showed that SOD2 polymorphisms could predispose to NIHL by exerting variable local tissue antioxidant roles, whereas Wang et al.^14^ only showed a weak association between SOD2 polymorphisms and NIHL. The current individual studies provide limited information and do not produce a convincing conclusion. Therefore, in this study, a meta-analysis with a relatively large sample was conducted in order to generate a more reliable conclusion regarding the relationship between SOD2 C47T polymorphism and NIHL.

## Methods

### Literature search, selection, and data collection

Articles investigating SOD2 and NIHL that were published in PubMed and Web of Science before December 2014 were included in this meta-analysis. The following search terms were used: superoxide dismutase, SOD2, polymorphism, polymorphisms, variation, variations, genotype, noise induced hearing loss, noise-induced hearing loss, and NIHL. Studies that met the following criteria were included: (1) full-text, English-written studies; (2) complete case-and-control data about the relationship between SOD2 polymorphism and NIHL; (3) sufficient data to infer the results; and (4) control group genotypes in Hardy–Weinberg Equilibrium (HWE). HWE was tested by the chi-squared test, and when a *p*-value of more than 0.05 was observed, the control group genotypes were consistent with HWE.

In this study, two investigators independently collected data from each eligible article. The data comprised first author, year of the publication, origin country, ethnicity, number of cases, and number of controls. Through checking between the two investigators, a final set of data was determined.

### Quality assessment

Study quality was evaluated by the Newcastle-Ottawa Quality Assessment Scale for case–control studies,^15^ in which the quality of the selected trials was determined on the basis of selection of the study groups (0–4 points), comparability of the study groups (0–2 points), and ascertainment of the outcome of interest (0–3 points).

### Data analysis

The association between C47T polymorphism in SOD2 gene and NIHL susceptibility was estimated under all genetic models. Five comparison models for C47T polymorphism were evaluated: an allele model (T *vs*. C), a co-dominant model (TT *vs*. CC and CT *vs*. CC), a dominant model (TT + CT *vs*. CC), and a recessive model (TT *vs*. CT + CC).

For the meta-analysis, pooled odds ratios (ORs) and 95% confidence intervals (CIs) were calculated using a fixed effects model or random effects model. The chosen model was based on the results of a heterogeneity test, which employed a previously described, *I*^2^ test statistics.^16^ If *I*^2^ was greater than 50%, a random effects model was used according to the DerSimonian and Laird method; otherwise, a fixed effects model was used according to the Mantel-Haenszel method.

Publication bias was tested using Begg's funnel plot and Egger's test.^17^ If the funnel plot was asymmetrical and Egger's test reported a *p*-value lower than 0.05, a publication bias likely existed.

All of analyses were performed using Stata version 12.0 software (Stata Corporation – College Station, TX, United States).

## Results

### Search results and study characteristics

**Figure 1:**
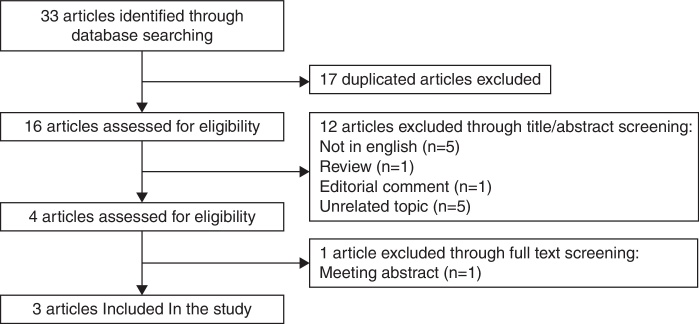
Flow chart of study inclusion.

The final search, which took place on December 31, 2014, resulted in the retrieval of 33 articles. The majority of the articles were excluded due to the fact that the study was about an unrelated topic, the articles were not in English, or the article was a duplicate, review, or commentary article, resulting in a total of four included articles. After a study which was a meeting abstract was omitted, three articles remained. Thus, three articles were finally included^6^, ^14^, ^18^ in this meta-analysis, comprising 1094 subjects, 407 of whom had NIHL. The review process is depicted in Fig. 1, which follows previously published reporting recommendations.^19^ Of the studies, two were conducted in the People's Republic of China and one was from Italy. All the genotype frequencies in control populations were in WHE agreement (Table 1).

**Table 1:** Characteristics of included studies.

Author	Year	Country	Ethnicity	Sample size (case/control)	Case (CC/CT/TT)	Control (CC/CT/TT)	*p*_HWE_
Fortunato et al.	2004	Italy	White	61/29	14/33/14	4a/19/6	0.08
Liu et al.	2010	China	Chinese	201/202	8/55/138	3/38/161	0.66
Li et al.	2014	China	Chinese	145/456	3/36/106	6/120/330	0.18

*p*_HWE_, *p*-value for Hardy–Weinberg equilibrium (HWE) test in each control group.

### Quality assessment of the included studies

The NOS for assessing the quality of case–control studies was shown in Table 2. All of the studies were identified as relatively high-quality because the total score was higher than 7.

**Table 2:** Quality assessment of included studies.

Author	Selection				Comparability		Exposure			Summary
	Is the case definition adequate?	Representativeness of the cases	Selection of controls	Definition of controls	Ethnicity	Age	Ascertainment of exposure	Same method of ascertainment for cases and controls	Non-response rate	
Fortunato et al.										4.1.3
Liu et al.										4.2.3
Li et al.										3.2.3

### Overall and subgroup meta-analysis results

**Figure 2:**
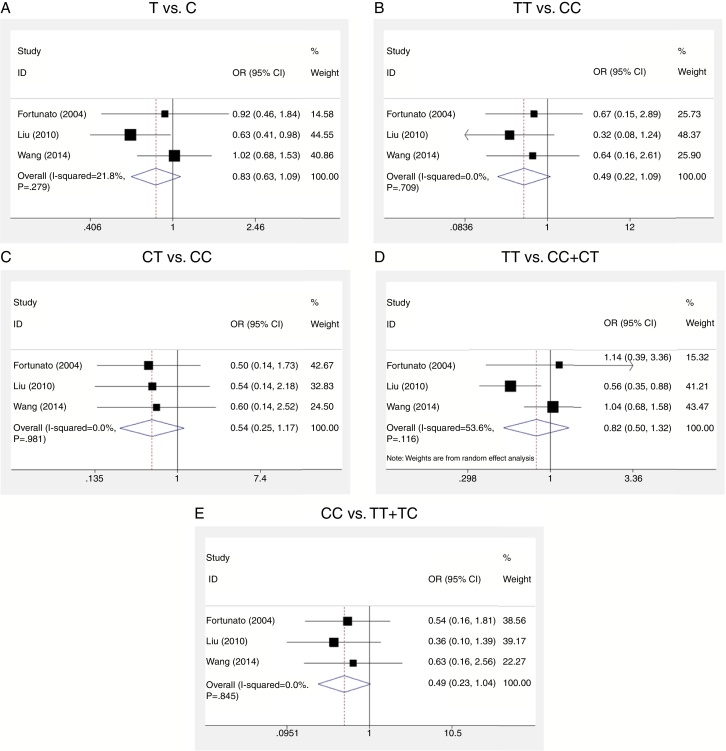
Forest plots regarding the association between superoxide dismutase 2 (SOD2) C47T polymorphism and noise induced hearing loss (NIHL) under all the genetic models using fixed effects or random effects models. Allele model, T *vs*. C (A); co-dominant model, TT *vs*. CC (B); CT *vs*. CC (C); recessive model, TT *vs*. CC + CT (D); dominant model, CC *vs*. TT + TC (E). CI, confidence interval; OR, odds ratio.

O meta-analysis and subgroup meta-analysis were performed based on ethnicity. The detailed results of the meta-analysis are shown in Table 3. Regarding overall meta-analysis, no significant association was observed under all genetic models (allele model: T *vs*. C, OR = 0.83, 95% CI = 0.63–1.09; dominant model: CC *vs*. TT + TC, OR = 0.49, 95% CI = 0.23–1.04; co-dominant model: TT *vs*. CC, OR = 0.49, 95% CI = 0.22–1.09; CT *vs*. CC, OR = 0.54, 95% CI = 0.25–1.17; recessive model: TT *vs*. CC + CT, OR = 0.82, 95% CI = 0.50–1.32; Fig. 2). When the subgroup analysis was categorized into Chinese and white ethnicities, a significant association was only observed between C47T polymorphism of the SOD2 gene and NIHL risk in the recessive model (TT *vs*. CC + CT, OR = 0.77, 95% CI = 0.42–1.41) in Chinese subjects (Table 2).

**Table 3:** Subgroup analysis based on ethnicity for all genetic models.

Ethnicity	No. of studies	Sample size (case/control)	T *vs*. C		TT *vs*. CC		CT *vs*. CC		TT *vs*. CC + CT		TT + CT *vs*. CC	
			OR (95% CI)	*p*	OR (95% CI)	*p*	OR (95% CI)	*p*	OR (95% CI)	*p*	OR (95% CI)	*p*
Chinese	2	346/658	0.82 (0.61–1.10)	0.12	0.43 (0.17–1.11)	0.48	0.57 (0.21–1.54)	0.92	0.77 (0.42–1.41)^a^	0.04^b^	0.46 (0.18–1.19)	0.58
White	1	61/29	0.92 (0.46–1.85)	–	0.67 (0.15–2.89)	–	0.50 (0.14–1.73)	–	1.14 (0.39–3.36)	–	0.54 (0.16–1.81)	–
Overall	3	407/687	0.83 (0.63–1.09)	0.28	0.49 (0.22–1.09)	0.71	0.54 (0.25–1.17)	0.98	0.82 (0.50–1.32)^a^	0.12	0.49 (0.23–1.04)	0.85

OR, odds ratio; CI, confidence interval.

*p*-Value for heterogeneity test; If *p* > 0.1, ORs were calculated using a fixed effects model, otherwise the random effects model was used.

a ORs were calculated using the random effects model.

b Significant association was observed.

### Publication bias

**Figure 3:**
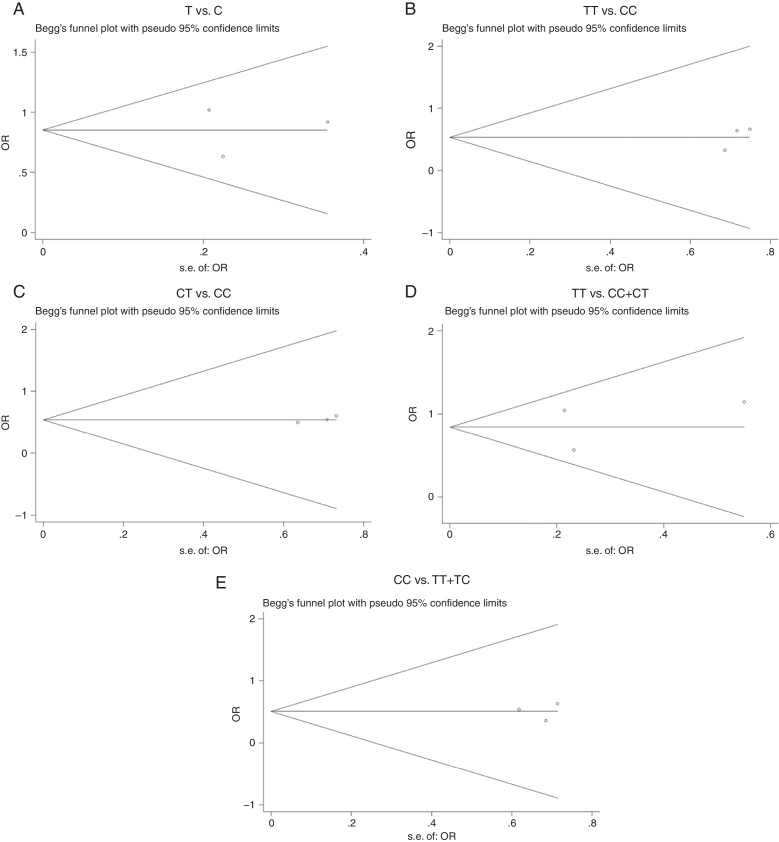
Begg's funnel plot for the superoxide dismutase 2 (SOD2) C47T polymorphism and noise induced hearing loss (NIHL). Allele model, T *vs*. C (A); co-dominant model, TT *vs*. CC (B); CT *vs*. CC (C); recessive model, TT *vs*. CC + CT (D); dominant model, CC *vs*. TT + TC (E). logor, odds ratio logarithm; s.e., standard error.

The results of Begg's funnel plot (Fig. 3 and Table 4) and Egger's test (Table 4) showed no publication bias for the allele model (T *vs*. C, *p* = 1.00), dominant model (CC *vs*. TT + TC, *p* = 0.952), co-dominant model (TT *vs*. CC, *p* = 0.306; CT *vs*. CC, *p* = 0.215), or recessive model (TT *vs*. CC + CT, *p* = 0.832).

**Table 4:** Publication bias using Begg's test and Egger's test under for all genetic models.

	T *vs*. C	TT *vs.* CC	CT *vs*. CC	TT *vs*. CC + CT	TT + CT *vs*. CC
Begg's test	1.00	0.296	0.296	1.000	1.000
Egger's test	1.00	0.306	0.215	0.832	0.952

## Discussion

Reactive oxygen species (ROS) play a key role in the underlying mechanisms of cochlear damage induction under various pathological conditions. Superoxide, which can form the highly toxic peroxynitrite, is readily generated in the inner ear following acoustic overstimulation.^20^ SOD is an enzyme involved in the regulation of superoxide levels by converting superoxide to hydrogenperoxide. Localization of SOD2 in the cochlea has been reported and absence of SOD2 has been shown to lead to increased hearing loss related to acoustic trauma.^14^, ^18^, ^21^, ^22^ In addition, auditory dysfunction due to noise exposure is attenuated by SOD2 application. Furthermore, transgenic mice overexpressing SOD2 were protected against aminoglycoside-induced hearing loss, which is also mediated by ROS.^23^ To date, several studies have explored the relationship between the SOD2 C47T polymorphism and NIHL susceptibility. For examples, Fortunato et al.^6^ previously showed that SOD2 polymorphisms could predispose to NIHL by exerting variable local tissue antioxidant roles, whereas Wang et al.^14^ only showed a weak association between SOD2 polymorphisms and NIHL. However, the protective effects of SOD2 remain controversial.

The present study analyzed the data from three studies that included a total of 407 NIHL cases and 687 controls. No significant associations between C47T polymorphism and NIHL were observed in all models. However, when subgroup analysis based on ethnicity was performed, a significant association was observed between the SOD2 C47T polymorphism and NIHL in a recessive model (TT *vs*. CC + CT, OR = 0.77, 95% CI = 0.42–1.41) in the Chinese population. There was no significant association in any other genetic models in the Chinese population. To the best of the author's knowledge, this was the first meta-analysis investigating the association between the SOD2 C47T polymorphism and NIHL susceptibility.

Moreover, fixed-effects or random-effects models were used in the analysis of the studies based on heterogeneity testing. Two study exhibits a substantial heterogeneity for the overall analysis. However, according to subgroup analysis based on ethnicity, no significant heterogeneity was observed. Therefore, further exploration of the risk factors for this condition is needed.

The present study has some limitations. For instance, the sample size used in this meta-analysis was insufficient, especially for the subgroup analysis based on ethnicity. Moreover, there was a lack of case–control data adjustment according to detailed individual information, such as age, sex, and lifestyle. The third limitation is that the exact molecular basis of the association between SOD2 C47T polymorphism and NIHL risk is still not clear at present and requires further investigation.

## Conclusion

Despite these limitations, the present meta-analysis suggested that SOD2 C47T polymorphism is significantly associated with an increased risk of NIHL in the Chinese population. Nevertheless, additional, larger and well-designed studies are needed to confirm this association.

## Conflicts of interest

The authors declare no conflicts of interest.
